# Experiences of patients with metastatic colorectal cancer participating in a supervised exercise intervention during chemotherapy

**DOI:** 10.1007/s00520-024-09101-1

**Published:** 2025-01-08

**Authors:** Calvin G. Brouwer, Marieke R. ten Tusscher, Bente M. de Roos, Elske C. Gootjes, Tineke E. Buffart, Kathelijn S. Versteeg, Isa H. Mast, Mirte M. Streppel, Inge M. Werter, Anne M. May, Henk M. W. Verheul, Laurien M. Buffart, on behalf of the AMICO Consortium, Jeanine M. L. Roodhart, Miriam L. Wumkes, Dirkje W. Sommeijer, Geert-Jan Creemers, Hans-Martin M. B. Otten, Annette van Zweeden, Aart Beeker, Johan J. B. Janssen, Karen Bolhuis

**Affiliations:** 1 Department of Medical BioSciences (HP 928), Radboud University Medical Center, Geert Grooteplein Zuid 10, 6525GA Nijmegen, The Netherlands; 2 Department of Medical Oncology, Radboud University Medical Center, Nijmegen, The Netherlands; 3 Department of Medical Oncology, Amsterdam University Medical Centers, Amsterdam, The Netherlands; 4 Department of Medical Oncology, Amphia Hospital, Breda, The Netherlands; 5 Department of Medical Oncology/Internal Medicine, Rijnstate Hospital, Arnhem, The Netherlands; 6 Department of Epidemiology, Julius Center for Health Sciences and Primary Care, University Medical Center Utrecht, Utrecht University, Utrecht, The Netherlands; 7 Department of Medical Oncology, Erasmus MC Cancer Institute, Erasmus Medical Center, Rotterdam, The Netherlands

**Keywords:** Exercise, Physical activity, Systemic treatment, Treatment tolerability, Qualitative study

## Abstract

**Purpose:**

Patients with metastatic colorectal cancer (mCRC) undergoing systemic treatment often experience toxicities. Although exercise may improve physical fitness and quality of life and counteract treatment toxicity, knowledge in patients with mCRC is limited. The ongoing randomized controlled AMICO trial evaluates the effects of supervised exercise on clinical outcomes. The present qualitative study was a pre-planned part of this trial aiming to capture adherence, satisfaction, and perceived effects of exercise among patients with mCRC.

**Methods:**

Patients with mCRC receiving first-line systemic treatment were randomized (1:1:1) to a control group or one of two supervised exercise arms including continuous aerobic exercise with either resistance exercises or high-intensity interval training. Semi-structured interviews with patients in the exercise arms were transcribed verbatim and thematically analyzed. Descriptive data on adherence (exercise logs) and satisfaction (questionnaire) was collected to complement and contextualize the qualitative findings.

**Results:**

Twenty-one patients were interviewed. Median exercise attendance was 67% [IQR 35–91], and the median satisfaction score was 8 [IQR 8–9] out of 10. Patients valued the guidance and knowledge of the physical therapist and expressed interindividual preferences regarding training content. Patients experienced that exercise improved their physical and mental wellbeing and helped them to endure treatment. Perceived exercise barriers were treatment toxicity, physical problems, and hospital appointments. Perceived exercise facilitators included adequate tailoring and internal or external motivation.

**Conclusion:**

Patients with mCRC appreciated exercise during systemic treatment and perceived several beneficial effects, both physically and mentally. Exercise attendance varied and barriers were mainly treatment- and disease-related.

**Trial registration:**

Clinical trial.GOV ID: NCT04754672.

Date of registration: 04–12-2020.

**Supplementary Information:**

The online version contains supplementary material available at 10.1007/s00520-024-09101-1.

## Introduction

Colorectal cancer is the second leading cause of all cancer-related deaths [1]. Over 50% of patients develop distant metastases [2]. Current median survival rates for patients with metastatic colorectal cancer (mCRC) is 24 to 30 months, highly dependent on mutation status [3]. First-line systemic treatment of most commonly occurring mismatch repair proficient mCRC generally includes fluoropyrimidines combined with oxaliplatin and/or irinotecan and targeted agents [4]. This is often associated with toxicities including myelosuppression, gastrointestinal toxicity, neuropathy, hand-foot syndrome, and fatigue [5, 6]. It has been reported that over 40% of patients with mCRC require toxicity-induced treatment modifications within the first 3 months of treatment, and around 30% are admitted to the hospital due to chemotherapy-related toxicity [5, 7]. This has been shown to reduce treatment outcome [8]. Mitigating toxicity is therefore crucial.

Exercise has been suggested as a non-pharmacological strategy to counteract systemic treatment toxicity in patients with (m)CRC [9]. Clinical guidelines recommend exercise to be part of standard care for patients with cancer [10] due to its beneficial effects on physical fitness and functioning, fatigue, and quality of life (QoL) [11–13]. However, most previous exercise trials have focused on patients with early-stage cancer. Despite the increasing number of studies reporting that exercise is also feasible and potentially beneficial for physical function and fatigue in patients with advanced cancer [14], there is limited knowledge on patients with mCRC. One small (*n* = 30) study in patients with mCRC found adequate session attendance, no serious adverse events, and positive effects of a multimodal exercise program on chemotherapy-related neuropathy [15], but adequate information on exercise adherence and patient experiences is lacking.

In the evaluation of complex interventions like exercise, the conduct of a qualitative study concurrently with a randomized controlled trial can provide a deeper understanding of experiences with delivering and receiving the intervention and with potential pathways underlying the effects [16]. Furthermore, information obtained from qualitative studies can help to guide future implementation in daily clinical practice [16].

The Aerobic Fitness or Muscle Mass training to Improve Colorectal cancer Outcomes (AMICO) trial was launched to evaluate the effectiveness of two different exercise interventions during systemic treatment versus usual care on chemotherapy treatment modifications in patients with mCRC. The current study was conducted as part of the AMICO trial and aimed to qualitatively examine adherence, satisfaction, and perceived effects of patients with exercise during systemic treatment. Descriptive data on these measures was collected to complement and contextualize qualitative data.

## Methods

### Design

The ongoing randomized controlled AMICO trial includes three arms: aerobic exercise combined with resistance exercise (AE + RE), aerobic exercise combined with high-intensity interval training (AE + HIIT), and a usual care (UC) control group (NCT04754672). The study was approved by the Medical Ethics Committee of East Netherlands (METC2020-6867) and institutional review boards of all participating hospitals.

### Patient eligibility and enrollment

In the AMICO trial, patients are recruited from fourteen Dutch hospitals. Patients are eligible when they are aged ≥ 18 years, diagnosed with mCRC and scheduled for first-line doublet (e.g., CAPOX or FOLFIRI) or triplet chemotherapy (e.g., FOLFOXIRI) with or without targeted agents, and have an estimated life expectancy > 6 months. Patients are excluded if they are unable to perform basic daily life activities (e.g., walking, biking) and have cognitive disorders, severe emotional instability, disabling co-morbidities hampering physical exercise or serious cardiovascular or cardiopulmonary conditions limiting exercise safety, or already participating in structured moderate-to-vigorous aerobic and/or resistance exercise ≥ 2 times per week comparable to the intervention. Potentially eligible patients are briefly informed about the study by their treating physician and subsequently contacted by the study coordinator. Patients who do not want to participate in the study are asked to provide a reason if they are willing to give one. Patients provide written informed consent prior to baseline assessments after which they are randomized and stratified by age (≤ 70 vs. > 70), previous chemotherapy treatment (yes vs. no), and type of chemotherapy (CAPOX vs. FOLFIRI vs. FOLFOXIRI). Randomization (1:1:1) is concealed within a cloud-based clinical data management platform, CASTOR®, using block randomization with block sizes varying between 6 and 9. Due to the nature of the intervention, patients and physical therapists are not blinded to group allocation.

### Exercise interventions

Both exercise interventions include two 60-min sessions per week, supervised by a local physical therapist specialized in oncology. The intervention starts after baseline assessment, which takes place before the first or second chemotherapy cycle and lasts throughout the treatment period with a maximum of 6 (CAPOX) or 8 (FOLFIRI/FOLFOXIRI) treatment cycles. Most exercise sessions are performed in small groups. Both interventions include 15- to 20-min moderate-intensity (Borg 13–14 “somewhat hard” [17]) continuous aerobic exercise on a cycle ergometer or treadmill.

In the AE + RE arm, continuous aerobic exercise is combined with resistance exercises, targeting 6 large muscle groups in 2 sets of 10 repetitions at 70–80% maximum workload, estimated using an indirect one repetition maximum (1RM) test. To ensure adequate training load over time, tests are repeated every 3–4 weeks aligned with the chemotherapy cycles.

In the AE + HIIT arm, continuous aerobic exercise is combined with 25 min of HIIT on a cycle ergometer. Three different interval sessions are offered alternatingly and are based on protocols previously used in patients with cancer: 4 × 4 min with 3-min active recovery [18]; 6 × 3 min with 1.5-min active recovery [19], and 7 × 2 min with 2-min active recovery [20]. The intensity of the intervals ranges from 85 to 95% of the estimated maximum heart rate, adjusted to Borg 16–18 (“hard–very hard”) [17]. Active recovery included light-intensity cycling (Borg < 12).

Patients in both exercise arms are asked to perform a third exercise session from home at moderate intensity for 30 min (e.g., walking or cycling).

### Data collection and analyses

For this study, both qualitative and descriptive data were used. Semi-structured interviews were held using an interview guide with open-ended questions (Supplementary file 1) to capture patients’ reasons for (non)adherence to, satisfaction with, and perceived effects of the exercise program. The semi-structured interviews were carried out in person, via video call or telephone by two researchers (CB and MT) directly after completion of the exercise intervention and transcribed verbatim. Interviewing was continued until data saturation. Thematic analysis was performed in the original Dutch language and coded in six phases [21] using ATLAS.ti web (version 23.3.4.28863):

1. Familiarization with the data by reading and re-reading transcripts of two interviews independently (MT, CB), after which a coding strategy was discussed to enhance uniform coding [22].
2. Data-driven, independent open coding of the transcripts of subsequent interviews by one investigator and reviewed by the other (MT, CB), until no new codes emerged.
3. Grouping codes into sub- and main themes, that were discussed until consensus (MT, CB). A third investigator (LB) was consulted when needed.
4. Reviewing and refining (sub)themes until the themes reflected the essence of the complete dataset (MT, CB, LB).
5. Providing names and definitions to all themes which captured the underlying significance. To illustrate the themes, relevant quotes (indicated with Q) were selected and translated into English by the investigators.
6. Answering the research questions using the themes from the previous phases.

We collected descriptive data on patients’ demographic and clinical characteristics through baseline questionnaires and medical records. Exercise adherence was assessed using exercise logs, where physical therapists recorded session attendance, reasons for non-attendance, exercise dose modifications, and any protocol adjustments. Exercise adherence was defined as the proportion of prescribed sessions attended and the exercise relative dose intensity (ExRDI), representing the percentage of exercise dose intensity during the performed sessions relative to the planned intensity and sessions [23].

Following the intervention, patients rated their satisfaction with the guidance of the physical therapist and the intervention’s impact on specific outcomes (physical fitness, QoL, mental wellbeing, and treatment tolerability) on a 0–10 scale. This data was summarized using medians and interquartile ranges (IQR) or numbers and proportions.

## Results

**Fig. 1:**
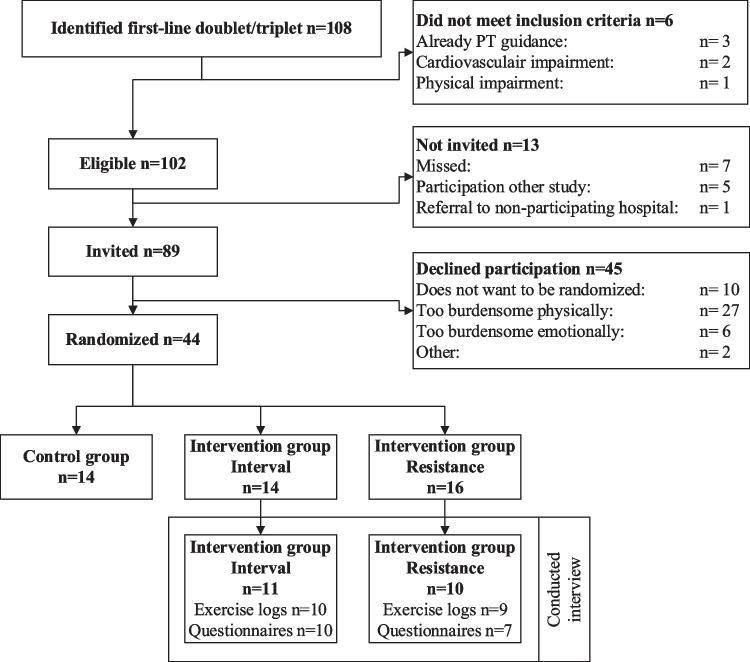
Flowchart of patient enrolment

Between March 2021 and December 2023, 89 patients were invited for the AMICO trial, of whom 44 (49%) participated. The main reasons for non-participation were not wanting to be randomized and too much physical or emotional burden at the time of inclusion (Fig. 1). In December 2023, 31 patients had completed the study of whom 21 were randomized into an exercise arm (10 AE + RE; 11 AE + HIIT) and interviewed. Of these 21 patients, 2 ended the intervention prematurely due to progression (both after 9 weeks), and 1 due to dissatisfaction with the intervention (after 8 weeks). Two patients had a good response after 8 and 12 weeks of systemic treatment, respectively, and consequently, the systemic treatment and coinciding exercise intervention finished. Exercise logs were available for 19 patients, of which 2 were incomplete. Questionnaires on satisfaction were available for 17 patients. The average age of the patients was 63.7 (SD 10.2) years and 71% received CAPOX (Table 1).

**Table 1:** Baseline characteristics of participants

	Total (*n* = 21)	Interval (*n* = 11)	Resistance (*n* = 10)
Age, mean (SD), years	63.7 (10.2)	62.5 (11.1)	65.0 (8.8)
Sex, *n* (%) female	10 (48)	5 (45)	5 (50)
Charlson comorbidity index elevated (≥ 1, primary malignancy excluded), *n* (%)	7 (33)	2 (18)	5 (50)
Prior cancer treatment, *n* (%)^a^			
- No prior treatment - (Neo) adjuvant chemotherapy	8 (40) 6 (30)	5 (45) 3 (27)	3 (33) 3 (33)
- Radiotherapy	1 (5)	1 (9)	0 (0)
- Surgery	5 (25)	2 (18)	3 (33)
Location primary tumor *n* (%)			
- Colon	13 (62)	6 (55)	7 (70)
- Rectum	8 (38)	5 (45)	3 (30)
Chemo type, *n* (%)			
- CAPOX	15 (71)	7 (64)	8 (80)
- FOLFIRI	1 (5)	1 (9)	0 (0)
- FOLFOXIRI	5 (24)	3 (27)	2 (20)
Married/living together, *n* (%) yes^a^	16 (80)	9 (90)	7 (70)
Education, *n* (%)^b^*			
Low	0	0 (0)	0 (0)
Intermediate	9 (47)	7 (78)	2 (20)
High	10 (53)	2 (22)	8 (80)

*SD* standard deviation,

^*^Low: primary, prevocational secondary education; Intermediate: senior general, pre university, or vocational education; High: higher or university education

^a^*n* − 1

^b^*n* − 2

### Adherence to the exercise interventions and the barriers and facilitators

**Fig. 2:**
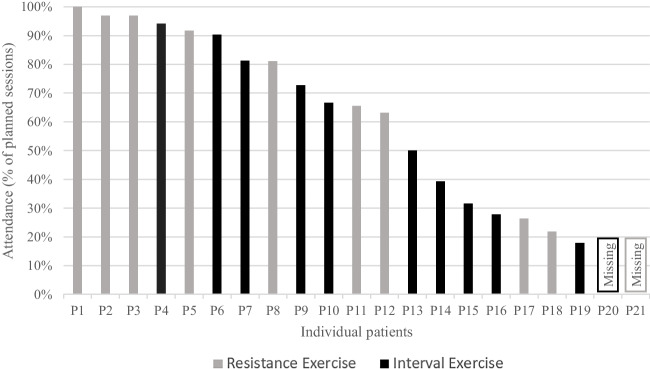
Exercise session attendance

Median exercise attendance was 67% (IQR 35–91%) of the planned sessions (Fig. 2), and median ExRDI was 61% (IQR 31–68%). Reasons for non-attendance were known for 111 (59%) missed sessions and included chemotherapy-related toxicities (26%), fatigue (21%), and other illness, not specifically related to cancer treatment (21%) (Supplemental Fig. 1A). Reasons for exercise dose adjustments were known for 106 (63%) adjusted sessions and included physical problems (43%, e.g., comorbidities or injuries), fatigue (26%), and chemotherapy-related toxicities (15%) (Supplemental Fig. 1B).

Patients mentioned various barriers to and facilitators of exercise adherence. Reported barriers included chemotherapy-related toxicities, physical problems, and illnesses. Symptoms such as diarrhea, nausea, dizziness, pain, and fatigue, particularly in the week following chemotherapy, were commonly reported barriers to exercise adherence (Table 2: Q1.1). The 46-h 5-fluoroucil infusion in patients receiving a 2-week chemotherapy cycle also reduced session attendance in the week after chemotherapy administration. Other reported barriers included difficulties with scheduling exercise sessions due to hospital appointments (Table 2: Q1.2), a lack of motivation or interest in exercise, and caregiving responsibilities (Table 2: Q1.3 and Q1.5.1).

**Table 2:** Main themes and subthemes emerged from interviews

Themes and subthemes	Selected exemplary quotes
1. Adherence—barriers	
1.1. Chemotherapy-related toxicities	1.1.1. “I had to miss several times… Because, either, I was not feeling well, or I had diarrhea due to the chemotherapy, and well, then I would cancel the session because, you know, it’s just not feasible.” (P14, male, 71 y) 1.1.2. “I was unable to attend the exercise sessions at the physical therapy practice due to the side effects of the chemotherapy.” (P17, male, 70 y) 1.1.3. “The second week went very well. But during the first week of chemotherapy cycle, I was too dizzy to be able to drive to the physical therapy practice.” (P13, male, 52 y)
1.2. Scheduling of appointments	1.2.1. “I had the chemotherapy on Thursday. And the physical therapist was available on Monday and Thursday, so I actually missed a session every week.” (P20, male, 60 y) 1.2.2. “In the second week, so when I was free from my infusions, I attended.” (P21, male, 58 y) 1.2.3. “During the week when I had chemotherapy, I would not go to the physical therapist, because on Mondays I had to get my blood drawn, on Tuesday I had to see the oncologist, and then Thursday was chemotherapy day again. And then I got hit by the chemotherapy on Friday, that made me go completely out again.” (P14, male, 71 y)
1.3. Lack of motivation to exercise	1.3.1. “When you are in that whole hospital mode, you know, you have little motivation to exercise.” (P13, male, 52 y) 1.3.2. “I think it’s just because I have always been anti-sports, to be honest.” (P15, female, 63 y)
1.4. Physical problems or illnesses	1.4.1. “Yes, the only downside is that my left knee is extremely bad… Well, that certainly affected the exercise training.” (P7, male, 78 y) 1.4.2. “In the beginning, I had quite a bit of trouble with bleeding hemorrhoids. Well, that is why I could not cycle properly.” (P6, female, 68 y)
1.5. Other circumstances	Responsibilities 1.5.1. “Monday and Friday are the days that I babysit the little ones. And then, I have to leave my husband there alone. And well, he will manage, but as a grandma, I always think I can do it better. Especially since there was a time when I had to pick up my granddaughter from school.” (P15, female, 63 y) Weather 1.5.2. “If it’s raining or if there’s a cold wind, I would not go outside because the chemotherapy has such a strong effect on my legs, arms, and face. I tried it once, and then I thought, well, I’m not doing this again.” (P3, female, 62 y)
**2.** **Adherence—facilitators**	
2.1. Weekly structure of supervised sessions	2.1.1. “If I did not have the appointment, maybe I would not have left the house in the beginning of my treatment. If I would have had to do it on my own, I think it would have been much more difficult.” (P8, female, 52 y) 2.1.2. “It’s also an encouragement (the appointment), to still go out the door and commit yourself to something.” (P20, male, 60 y)
2.2. Tailored exercise sessions	Physical problems: 2.2.1. “I cannot perform those squats with my thighs. It would give me a lot of pain. The physical therapist immediately removed that exercise and adjusted it for me.” (P12, female, 68 y) Chemotherapy cycle: 2.2.2. “During the exercises, the physical therapist also took into account when I had just received a chemotherapy infusion. The intensity was a bit lower. You simply have less strength, so you can do a bit less. And then the load was gradually built up again.” (P4, male, 75 y)
2.3. Positive experience with exercise	2.3.1. “Normally, I used to do a lot of sports. That was an outlet for me. Now, I could to do this twice a week.” (P13, male, 52 y) 2.3.2. “Afterwards, I went home with a happy feeling because I had accomplished something. That I did not take a step backwards, you know, that was a victory for me every time.” (P10, male, 53 y) 2.3.3. “I do not always feel like exercising, but I have learned that when I do it anyway, I feel good afterwards.” (P13, male, 52 y)
2.4. Social support	Personal: 2.4.1. “Sometimes I felt like skipping the session… But then my husband said, ‘No, you should go, come on.’ That really helped me.” (P18, female, 50 y) Professional: 2.4.2. “I have to say, the oncologist quite often asked how the exercise sessions were going, so that’s also nice.” (P10, male, 53 y) 2.4.3. “The physical therapist also gives good advice to be active, for example, go for a half-hour walks… And after the talk you feel very comfortable.” (P19, female, 42 y)
2.5. Home environment	2.5.1. “Fortunately, I had a bike at home, so, every now and then, I would cycle for about ten minutes at home. Then, I only had to walk out of the room to reach the toilet. That was nice.” (P14, male, 71 y)
**3.** **Satisfaction—expectations**	
3.1. Expectations prior to participation	Exceeded expectations: 3.1.1. “It was harder than I expected, they really wear you out during the exercise sessions.” (P3, female, 62 y) 3.1.2. “I thought I could never keep up with the exercise sessions, but I succeeded.” (P9, female, 76 y) No expectations: 3.1.3. “I just came open minded and let it happen. With all the things that come my way, I also did not know exactly what was going to happen.” (P7, male, 78 y) Positive expectations: 3.1.4. “Well, I expected that exercise would help to maintain my activities. So that I would do more exercises, than I would have done on my own. Yes, that worked out well.” (P13, male, 52 y)
**4.** **Satisfaction—non-contributors**	
4.1. Misalignment with personal preferences	Exercise frequency 4.1.1. “I found training twice a week to be too much because there were times I could not do it. Once a week would have been enough for me.” (P18, female, 50 y) Exercise intensity 4.1.2. “Well, in the beginning, it was really tough. The strength in my legs was completely gone. And then I had to get on that bike, and just lifting my leg was already a problem.” (P14, male, 71 y) Exercise type 4.1.3. “Well, I also do not really like strength training machines.” (P12, female, 68 y) 4.1.4. “It does get quite monotonous, of course, cycling twice a week for 18 weeks in a row.” (P10, male, 53 y) 4.1.5. “You can do squats in many different ways… It’s more effective for the training effect. And if I have to do the same training thirty times in a row, I know what I’m doing, but I think some variation would be more fun.” (P2, male, 66 y)
4.2. Confronting	Group sessions 4.2.1. Q1.2.3 “I happen to train at a physical therapy practice that only treats patients with cancer. It’s quite an experience, to hear all the stories of others.” (P2, male, 66 y) Physical deterioration 4.2.2. “The physical therapist also decided that the resistance could be reduced a bit. But then I got upset because I lost so much of my fitness, which was hard for me.” (P8, female, 52 y)
**5.** **Satisfaction—contributors**	
5.1. Alignment with personal exercise preferences	Exercise type: 5.1.1. “Normally, I always cycled indoors as well.” (P13, male, 52 y) 5.1.2. “There were a few exercises that I just really liked to do… Eventually, I found everything pleasant to do, and there was sufficient variety in exercises.” (P12, female, 68 y) Exercise Frequency 5.1.3. “Maybe you can train more often, maybe three sessions per week instead of two, especially during this cold winter period, and in case of bad weather when people tend to stay indoors more often.” (P20, male, 60 y) Exercise Intensity 5.1.4. “The level of effort that I would never impose on myself otherwise. If I were to exercise normally, I would not go that hard. Sometimes, that’s actually really good to do.” (P10, male, 53 y)
5.2. Exercise with peers in group sessions	5.2.1. “Because I got in touch with people who all, more or less, had to deal with the same thing. It felt very nice to support each other. I noticed that it cheered me up to talk to each other about certain things.” (P3, female, 62 y) 5.2.2. “I think it’s important to exercise at a specialized gym, because practically 90% of the people there all have the same problems. It’s nice that nobody questions when you come in and get on the bike wearing gloves or a scarf. I found that very pleasant.” (P3, female, 62 y)
5.3. Support of physical therapist	Personal and mental support: 5.3.1. “The conversations with the physical therapist and the advice the physical therapist gave me helped a lot.” (P18, female, 50 y) 5.3.2. “The physical therapist is incredibly good at providing personal support.” (P17, male, 70 y) 5.3.3. “I know that being able to share my experiences is very important to me, it refreshes me mentally.” (P5, female, 78 y) Knowledge: 5.3.4. “I understand why it is important to visit a physical therapist specialized in oncology, because they really know a lot more.” (P17, male, 70 y)
**6.** **Perceived effects—general**	
6.1. Effects difficult to interpret	6.1.1. “I do not have a comparison; I have not gone through the chemotherapy period without physical therapy. So, it’s challenging to evaluate the exact effect.” (P10, male, 53 y)
6.2. Training provided benefits despite low adherence	6.2.1. “I have only visited the physical therapist a few times, but these sessions helped me a lot.” (P19, female, 42 y) 6.2.2. “I only went a few times due to the intensity of the chemotherapy, but those few times really helped me being more physically active, and I’m happy about that.” (P18, female, 50 y)
**7.** **Perceived effects—physical wellbeing**	
7.1. Physical fitness	Positive: 7.1.1. “Well, it has definitely improved my overall fitness. I noticed that my fitness declined during the chemotherapy. And through those workouts, I am essentially building it up again. So, that is a positive experience.” (P20, male, 60 y) 7.1.2. “I felt that it makes me stronger. Yes, more endurance, definitely, yes.” (P9, female, 76 y) Negative: 7.1.3. “I noticed, for example, that I have lost some strength in my hands and arms, and well, I have to start exercising again.” (P8, female, 52 y) 7.1.4. “I do not really notice an immediate increase in my fitness after just practicing or exercising once.” (P21, male, 58 y)
7.2. Physical functioning	Walking: 7.2.1. “I noticed that I am better able to lift my bag, walk a bit longer in the city, feel less tired, and, I am walking faster, according to my daughter, I do not have the pace of an old woman anymore.” (P3, female, 62 y) Household chores: 7.2.2. “I still do my chores at home, my laundry, vacuum cleaning. And yes, maybe it is also thanks to the exercise sessions. For as long as I can, I will do the chores myself, and I do not want any help with that.” (P14, male, 71 y) Shopping: 7.2.3. “The things I do at home, I had to go grocery shopping, and if there is a case of beer in the car, and then I just pick it up like I always did.” (P7, male, 78 y)
7.3. More energy	7.3.1. “And when I went exercising again, I noticed that it is going better. I gained more energy and also more energy to undertake other activities.” (P20, male, 60 y) 7.3.2. “Exercise has helped me to gain more energy, and I can also notice the difference between the first time I came to the exercise session and now. That is a huge difference, and well, that makes me happy when I regain energy. So, it has brought me a lot.” (P6, female, 68 y)
**8.** **Perceived effects—mental wellbeing**	
8.1. Psychological	Positive: 8.1.1. “I always came back happy again.” (P13, male, 52 y) 8.1.2. “Yes, absolutely, because when I left, I felt satisfied, because I had done it again.” (P15, female, 63 y) Neutral: 8.1.3. “I did not really have much trouble with mental issues or feeling down or anything like that. Yeah, that’s just how I am.” (P4, male, 75 y)
8.2. Quality of life	Positive 8.2.1. “After the physical therapy, I felt stronger, I wanted to do something fun. So, it contributed to my quality of life.” (P18, female, 50 y) 8.2.2. “Well, in terms of my wellbeing, I was a physically active woman. And thankfully, I still am. I think that is partly due to the exercise sessions.” (P8, female, 52 y) Neutral 8.2.3. “I honestly do not know if it affected my quality of life. Every time after the chemotherapy, I did not feel well… I do not know if the physical therapy really contributed, I’m very honest about that.” (P15, female, 63 y) 8.2.4. “I do not think it affected my quality of life. I still have a lot to do at home, I have to take care of my wife.” (P16, male, 76 y)
8.3. Empowerment	Positive: 8.3.1. “I engage in some activities, like making music. I occasionally attend concerts and so. I usually manage to do so, and it gives me a goal. That motivates me. The physical therapy makes me feel that I can handle it. That’s definitely a positive point of the physical therapy.” (P11, male, 66 y) 8.3.2. “You gain more resilience, to go out and do things.” (P20, male, 60 y) 8.3.3. “I’m active six out of seven days, that definitely helps with my mental resilience and so on.” (P19, female, 42 y)
**9.** **Perceived effects—treatment side effects and tolerability**	
9.1. Side effects of treatment	Fatigue: 9.1.1. “The cancer treatment causes a lot of fatigue, so I tend to want to rest more. The danger is actually that you stop doing anything, not any or too little physical activity. The appointments with the physical therapist and the exercises encouraged me to go there, to exercise and to overcome fatigue.” (P11, male, 66 y) Chemotherapy tolerability: 9.1.2. “I am gaining more strength again. And that strength or fitness is needed to get rid of the junk that you get in your body.” (P2, male, 66 y) 9.1.3. “And I also think that is why I can better cope with the chemotherapy and the side effects. I do experience the side effects, but well, I do not let them bother me too much.” (P3, female, 62 y) Neuropathy: 9.1.4. “I had a bit of discomfort in my legs, a bit wobbly. It disappeared due to the exercise sessions.” (P4, male, 75 y) Comparison to previous treatment: 9.1.5. “The last time I had chemotherapy, we stopped after three cycles because I had so many side effects, and now I completed all six, so I noticed the difference… Previously, I was just tired all the time, and I preferred to lie on the couch all day to sleep. Now, that was a lot less. I am more active.” (P3, female, 62 y) 9.1.6. “Last year during chemotherapy, I was really lying at home like a sack of potatoes, waiting for time to pass. And now, it’s quite different.” (P9, female, 76 y)
9.2. Tumor reduction	9.2.1. “I am really curious what exercise does with the cancer itself, because the spot on my lungs is gone. Is the chemo doing that or is it my physical activity?” (P8, female, 52 y)
**10.** **Sustainability of effects**	
10.1. Knowledge gain	10.1.1. “And the gradual progression, I have certainly learned from that, and I could apply that again if necessary.” (P17, male, 70 y)
10.2. Future exercise perspectives	With physical therapist: 10.2.1. “It says something that, I have decided to keep going to the physical therapist twice a week, now the study has finished for me.” (P3, female, 62 y) 10.2.2. “I want to stay with the physical therapist because she has a lot of experience with relapses and complications.” (P12, female, 68 y) On their own: 10.2.3. “Well, I will continue to exercise, maybe in the gym or via another way, but I want to stay physically active to maintain my fitness level.” (P4, male, 75 y)

Reported facilitators of exercise intervention adherence included the scheduled appointment with the physical therapist, the weekly structure that it provided, and participating in an exercise study in general (Table 2: Q2.1). The ability of the physical therapist to tailor the content of the exercise training to physical problems was also reported as a facilitator of exercise adherence, even in the days after chemotherapy administration when experiencing the most chemotherapy-related toxicities (Table 2: Q2.2). Other reported facilitators included personal traits including perseverance and experiencing joy and fulfillment during exercise as motivators for attending subsequent sessions (Table 2: Q2.3). Support from the social environment, including encouragement from the spouse, physical therapist, and oncologist, also contributed to adherence (Table 2: Q2.4). Many patients reported that they were able to be physically active in their home environment, next to the supervised exercise sessions. Some patients preferred exercising at home over the supervised exercise sessions at the physical therapy practice if they experienced chemotherapy-related toxicities such as diarrhea (Table 2: Q2.5). Other patients mentioned that adherence to exercise from home exercise was affected by environmental factors like temperature or weather.

### Satisfaction with the exercise interventions

**Fig. 3:**
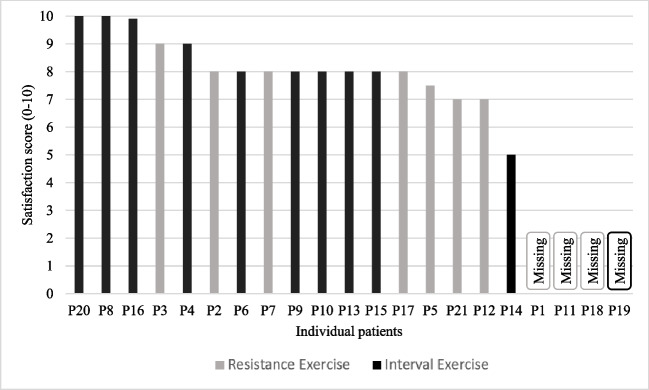
Satisfaction with the exercise intervention

The median overall satisfaction with the intervention was 8 [IQR 8–9] out of 10 (Fig. 3, Supplemental Table 1). Most patients appreciated participation in the intervention and were satisfied. However, some patients reported that the exercises were a bit too strenuous in combination with the treatment and/or that they would have preferred a lower training frequency or intensity (Table 2: Q4.1.1/2). Additionally, variation in exercise type was mentioned to influence satisfaction based on personal preferences, with some patients suggesting that more variation would improve the intervention (Table 2: Q4.1.3–5 and Q5.1.1/2).

For some patients, satisfaction was improved by group sessions as they appreciated contact with peers and discussing the disease without judgement (Table 2: Q5.2). However, the group sessions negatively impacted the satisfaction of others as they found it too confronting to be in regular contact with peers and face their illnesses (Table 2: Q4.2.1).

Patients were satisfied with the supervision of the physical therapist, particularly valuing the knowledge of the physical therapist about their medical status and how to adjust the exercises to chemotherapy-related toxicities. The opportunity to share experiences with someone who listens and offers feedback was perceived as an important aspect of the exercise sessions (Table 2: Q5.3).

### Perceived effects of the exercise interventions

Patients expressed several positive effects of the exercise intervention, although some found it difficult to mention the effects due to the lack of comparison. The median score of the extent to which the interventions contributed to physical fitness was 8 [IQR 7–9] out of 10; it was 7 [IQR 5–8] for QoL, 7 [IQR 5–8] for mental wellbeing, and 7 [IQR 6–8] for treatment tolerability (Supplemental Table 1). Positive effects were also reported by patients who had lower exercise adherence. Patients mentioned that the physical therapists’ guidance and support to be physically active motivated them, even when attending the supervised exercise sessions was not possible (Table 2: Q6.2). Improvements or maintenance in physical fitness were commonly reported effects, enabling patients to perform daily functional activities such as household chores and shopping, and experiencing increased energy for social events (Table 2: Q7.2). However, some patients did not notice the benefits on physical fitness. One patient from the AE + HIIT arm mentioned to have lost some strength in the upper extremities (Table 2: Q7.1.3).

Patients also perceived benefits in their mental wellbeing, including feelings of happiness and pride after completing an exercise session (Table 2: Q8.1.1/2). Patients expressed that exercise positively influenced their QoL and empowerment, because it enabled them to maintain physical activity levels and participate in social events during chemotherapy (Table 2: Q8.2/3). Some patients reported that QoL was not affected by the exercise intervention, because it was mainly influenced by other aspects such as daily caregiving tasks (Table 2: Q8.2.4).

Patients indicated that the exercise intervention helped them to better tolerate the chemotherapy toxicity. Two patients indicated feeling much better compared to their previous (adjuvant) chemotherapy treatment without an additional physical therapist–guided exercise intervention (Table 2: Q9.1.5/6). Additionally, patients learned how to perform exercises and to be physically active by themselves and expressed that this knowledge would enable them to incorporate exercises into their future daily routines. Patients reported to have planned to continue exercising either under the supervision of the physical therapist, at the gym, or by participating in other sports (Table 2: Q10.2).

## Discussion

This study examined adherence to, satisfaction with, and perceived effects of an exercise intervention during first-line systemic treatment in patients with mCRC. Results showed that almost half of patients with mCRC receiving systemic treatment were willing to participate in an 18-week exercise intervention, but adherence varied between patients, due to various individual barriers and facilitators. Additionally, we found that most participating patients were satisfied with the exercise interventions and the guidance by the physical therapist to ensure an adequately tailored intervention and support. Furthermore, patients perceived several beneficial effects on physical fitness and function, mental wellbeing, and treatment tolerability.

The overall adherence was acceptable, but there were considerable interindividual differences. The 67% attendance rate was in line with the 44–95% reported for other exercise interventions in patients with advanced cancer [24]. The ExRDI of patients receiving chemotherapy during advanced cancer is not well reported, but the 61% we found aligns with findings from a previous study in patients with metastatic breast cancer receiving an aerobic exercise intervention [25].

Patients in this study indicated that attendance was primarily influenced by chemotherapy-related toxicities, particularly impacting exercise participation in the week following chemotherapy administration. However, patients described that adequate tailoring of the exercise sessions to these toxicities or other physical problems facilitated adherence. Likewise, a “chemotherapy-periodized” exercise prescription, accommodating cyclical variations in chemotherapy administration and toxicity, has been shown to increase exercise attendance in patients with early-stage breast cancer [26]. While supervised exercise interventions have shown larger effects on physical fitness, fatigue, and QoL compared with unsupervised interventions [27], our results showed that home-based exercise sessions could be valuable next to supervised sessions, for example, when patients are unable to attend exercise sessions during continuous infusions or due to gastrointestinal toxicity. Other qualitative and survey studies also indicated that home-based exercise facilitated exercise adherence in patients receiving palliative treatment [28, 29]. Future studies should reveal if hybrid interventions, including both supervised and home-based exercise sessions, will improve adherence in patients with mCRC during chemotherapy.

While good overall satisfaction with the exercise interventions, the interindividual differences in preferences towards exercise intensity and type support further personalization of exercise programs (based on preferences, physical capabilities, and toxicities) to maintain or increase motivation and satisfaction. In line with a qualitative study of patients with esophageal cancer, patients were especially satisfied with the guidance by the physical therapists because of their knowledge of cancer and the treatment, sympathetic ear, and motivating role [30]. This created an environment in which patients felt safe to exercise and express themselves. Additionally, patients perceived group-based training as positive due to the additional support and recognition by peers. This is in line with other studies describing that group-based training has the potential to maintain motivation and increase coping with cancer [28, 31, 32].

The finding that patients with mCRC experienced positive effects on physical fitness and function, mental wellbeing, and treatment tolerability during systemic treatment adds to the results from previous qualitative studies in other cancer populations [31, 33], and the evidence from randomized controlled trials reporting the potential of exercise to improve physical functioning, fatigue, and QoL in patients with advanced cancer [14, 34]. The reported feelings of fulfillment, joy, and empowerment experienced by participating patients may explain the beneficial effect on QoL. Comparably, previous studies showed that exercise may change the perspective of fatigue towards a more positive and less frustrating perspective [31, 35]. Additionally, exercise may stimulate the feeling of being in control and personally contribute towards health, which can result in better psychological wellbeing [28, 31]. Still, some patients noted no improvement in their QoL despite being satisfied with the intervention content and experiencing beneficial effects on physical fitness. QoL encompasses many domains, including physical, mental, and social well-being, and exercise may differentially affect the domains across individuals [36]. Finally, the perception of enhanced coping with treatment toxicity resulting from improved physical functioning, fitness, and energy is in line with previously reported perceptions of patients that exercise helped them to better cope with cancer treatments by giving them both physical and psychological strength [37]. Considering that patients with mCRC often receive multiple lines of treatment [38, 39], the positive experiences of exercise during first-line treatment and the empowerment to maintain physical activity and fitness levels may have important consequences to better endure multiple treatments while maintaining functional independence and QoL.

Interestingly, the reported beneficial effects did not seem to be directly related to exercise intervention adherence, as some patients who completed fewer than ten sessions still reported benefit. Previous exercise trials have also suggested that lower exercise volumes or intensities may be sufficient to experience benefits on fatigue and QoL [40–42]. According to these patients, the few sessions with the physical therapists provided knowledge about the role and benefit of exercise, which motivated them to exercise in their home environment. Correspondingly, providing information about consequences for health and how to perform behaviors have been identified as techniques to improve the physical activity behavior of patients after cancer diagnosis [43, 44].

While data saturation was reached for the qualitative data, and contextualized with descriptive data on patient’s satisfaction and adherence, the generalizability of the descriptive data may be uncertain. Generalizability may also be hampered by participation bias given that patients who are interested in exercise are generally more willing to participate in exercise trials [45]. Another limitation arises from the utilization of exercise logs to register exercise dose modifications. Despite the simplicity of these logs, there was variation among physical therapists in documenting the reasons for adjustments in exercise frequency, intensity, and duration. This variance may have introduced uncertainty regarding the assessment of ExRDI and reasons for adjustments. Likewise, missing data in the satisfaction questionnaires may have also introduced information bias potentially resulting in over- or underestimation of the satisfaction. Finally, although interviews were conducted by investigators not directly involved in delivering the exercise intervention and some patients indicated feeling comfortable expressing dissatisfaction, we cannot dismiss the possibility of social desirability bias.

Our findings on the experiences of exercise during systemic treatment are a first step towards understanding the potential benefit of exercise programs to improve QoL and treatment tolerability in patients with mCRC. The experiences of exercise were not compared to a control group here, but are currently under investigation in the ongoing AMICO trial.

## Conclusion

Results of this study showed that participating patients with mCRC in an exercise program are satisfied with the intervention during first-line systemic treatment and experience positive effects on physical and mental well-being and treatment tolerability. Adherence to the exercise intervention varied and patients suggested that this may further be improved by tailoring the intervention to patients’ preferences, comorbidities, treatment-related toxicities, and treatment schedules.

## Supplementary Information

Below is the link to the electronic supplementary material.

Supplementary file1 (DOCX 26 KB)

## Data Availability

The datasets used and/or analyzed during the current study are available from the corresponding author on reasonable request.
