# Supplementary material for: Experiences of patients with metastatic colorectal cancer participating in a supervised exercise intervention during chemotherapy
Source: Support Care Cancer. 2025 Jan 8;33(2):82. doi: 10.1007/s00520-024-09101-1 (PMC11711133; doi:10.1007/s00520-024-09101-1)
Supplement: Supplementary file 1 — Supplementary file1 (DOCX 26 KB) [file 520_2024_9101_MOESM1_ESM.docx]

**Supplemental file 1: Interview guide**

1. Could you tell us about your experiences with the exercise intervention during the chemotherapy treatment?
   1. To what extent did the intervention meet your expectations prior to the start?
   2. Did you have other experiences with the exercise intervention?
2. How much did you adhere to the complete exercise intervention?
   1. To what extent were you able to (continue to) participate in the intervention? If not, why not?
      - - 1. Supervised training with the physical therapist
          2. Independent exercising the third-time each week
   2. What helped you to (continue to) participate in the exercise intervention?

Note: For the following questions, please use the provided answers to the satisfaction questionnaire.

1. You indicate that the intervention has contributed to your physical fitness with a … on a 10 point scale.
   1. How did you notice this? Can you provide examples?
   2. Or, can you explain why the intervention not or hardly contributed to this?
2. You indicate that the intervention has contributed to your quality of life with a … on a 10 point scale.
   1. How do you notice this? Can you provide examples?
   2. Or, can you explain why the intervention not or hardly contributed to this?
3. You indicated that the intervention has contributed to your mental wellbeing with a … on a 10 point scale.
   1. How did you notice this? Can you provide examples?
   2. Or, can you explain why the intervention not or hardly contributed to this?
4. You indicated that the intervention has contributed to tolerating chemotherapy with a… on a 10 point scale.
   1. How do you notice this? Can you provide examples?
   2. Or, can you explain why the intervention not or hardly contributed to this?
5. To what extent did the exercise intervention align with your personal preferences and needs?
   1. How could the exercise intervention better align with your personal preferences?
6. How do you think the exercise intervention could be improved?
   1. How could the training sessions be improved?
7. Do you have any further comments on (your participation in) the exercise intervention?
8. Do you plan to continue training, like in the exercise intervention, at home or in the gym?

**Supplemental figure 1A: Reasons for non-attendance of training sessions**

**Supplemental figure 1B Reasons for adjustments of exercise sessions**

**Supplemental table 1: Results from the satisfaction questionnaire**

|  | Totaal (n=17) | AE+HIIT (n=10) | AE + RE (n=7) |
| --- | --- | --- | --- |
| Training program, Median [IQR] | 8 [8-9] | 8 [8-9.7] | 8 [7.3-8] |
| *Training sessions contributed to:* |  |  |  |
| Physical fitness, Median [IQR] | 8 [7-9] | 8 [7-9] | 7 [5.5-8] |
| Quality of life, Median [IQR] | 7 [5-8] | 7 [7-8.8] | 6 [4.5-7.5] |
| Mental wellbeing, Median [IQR] | 7 [5-8] | 7 [7-8] | 8 [4.5-8] |
| Chemotherapy treatment tolerability, Median [IQR] | 7 [6-8] | 7 [7-9.8] | 6 [4-8] |

IQR: Interquartile range
